# Prevalence of species of yellow, purple and green microbial complexes in endo-perio lesions: a systematic review

**DOI:** 10.1590/1807-3107bor-2024.vol38.0048

**Published:** 2024-06-24

**Authors:** Diego José GAMBIN, Filipe Colombo VITALI, Keli Adriana Silvestre CASANOVA, João Paulo DE CARLI, Ricardo Ruiz MAZZON, Brenda Paula Figueiredo de Almeida GOMES, Micheline Sandini TRENTIN, Thais Mageste DUQUE

**Affiliations:** (a) Universidade de Passo Fundo – UPF, School of Dentistry, Passo Fundo, RS, Brazil.; (b) Universidade Federal de Santa Catarina – UFSC, Department of Dentistry, Florianopolis, SC, Brazil.; (c) Universidade de Passo Fundo – UPF, School of Dentistry, Department of Oral Medicine and Prosthodontics, Passo Fundo, RS, Brazil.; (d) Universidade Federal de Santa Catarina – UFSC, Department of Microbiology, Immunology and Parasitology, Florianopolis, SC, Brazil.; (e) Universidade Estadual de Campinas – Unicamp, Piracicaba Dental School, Department of Restorative Dentistry, Piracicaba, SP, Brazil.; (f) Universidade de Passo Fundo – UPF, School of Dentistry, Department of Periodontics, Passo Fundo, RS, Brazil.

**Keywords:** Dental Pulp, Microbiology, Periodontal Diseases, Systematic Review

## Abstract

This review aimed to determine the prevalence of species of yellow, purple and green microbial complexes in root canals (RC) and periodontal pockets (PP) of teeth with endodontic-periodontal lesions. For this purpose, two reviewers searched the literature up to January 2022. Studies reporting the prevalence of species of the yellow, purple and green microbial complexes in teeth diagnosed with endodontic-periodontal lesions were included. The risk of bias of the included studies was assessed using the 14 criteria from the NIH Quality Assessment Tool. Of 1,611 references identified in the initial search, only four studies were eligible and included in the qualitative analysis. The profile and prevalence rates of bacterial species in RC and PP varied among the included studies: levels of *Agregatibacter actinomycetemcomitans* (12% RC, 58% PP), *Capnocytophaga granulosa* (10% RC, 35% PP), *Capnocytophaga sputigena* (15-70% RC, 0-30% PP), *Streptococcus mitis* (30% RC, 35% PP), *Streptococcus sanguinis* (30% RC, 35% PP), and *Veillonella parvula* (70% RC, 50% PP) were identified. The high methodological heterogeneity prevented grouping and quantitative analysis of data. The risk of bias was considered ‘moderate’ for all studies. The included studies identified the presence of seven bacterial species belonging to the yellow, purple, and green microbial complexes in RC and PP, but with different prevalence rates. Future clinical studies are encouraged to investigate the presence and role of these species in the occurrence and development of endodontic-periodontal lesions.

## Introduction

Endodontic-periodontal lesions are characterized by independent or concomitant pathological involvement of the pulp and periodontal tissues in the same tooth.^1, 2^ These lesions continue to pose a challenge to the clinician in terms of etiology, diagnosis, and therapeutic approach.^2^ When not correctly diagnosed and treated, endodontic-periodontal lesions can lead to acute conditions in the dental complex or, in more complex cases, to tooth loss.^3^ Therefore, the etiopathogenesis of these lesions needs to be adequately investigated in the literature.^4^

Clinically, the correlation between endodontic and periodontal lesions makes it difficult to establish the most appropriate treatment.^1^ Based on this, Simon and collegues^1,5^ offer a classification of endodontic-periodontal lesions based on their possible etiology, diagnosis, and prognosis of treatment, as follows: primary endodontic lesions; primary periodontal lesions; primary endodontic lesions with secondary periodontal involvement; primary periodontal lesions with secondary endodontic involvement; and true combined lesions. Although there are other classifications proposed for endodontic-periodontal lesions,^6,7^ the classification proposed by Simon and colleagues is the most accepted and currently used in terms of the clinical and radiographic aspects.^1,5^

Microorganisms play a fundamental role in the development and maintenance of inflammatory processes that affect the pulp and periodontal tissues and can cause great tissues destruction due to the release of their toxic by-products.^8^ The microbiological profile of endodontic-periodontal lesions is still not completely defined.^2, 9^ Microbiological studies have shown that the microbiota and inflammatory profile in root canals and periodontal pockets adjacent to the roots of teeth with these lesions are similar,^10,11^ it is suggested that periodontal pockets can be an important source of infection for the pulp tissue.^12^

The bacterial species associated with periodontal lesions are distributed into five complexes: red, orange, yellow, purple, and green.^13,14^ Red and orange complexes are characterized by the predominance of anaerobic gram-negative bacteria.^9^ The red complex is composed of three bacterial species: Porphyromonas gingivalis, Treponema denticola, and Tannerella forsythia, which have been associated with periodontal disease.^9^ The orange complex include the Fusobacterium and Campylobacter species Parvimonas micra, Prevotella intermedia, Prevotela nigrescens, Streptococcus constellatus, and Eubacterium nodatum.^9^ Species of this complex are primary and late colonizers of dental biofilm, being responsible for modulating the biofilm and thus favor the aggregation and nutrition of strictly anaerobic species that perpetuate in periodontal pockets.^13^

Yellow, purple, and green microbial complexes comprise a set of aerobic gram-positive and anaerobic gram-negative species in the form of cocci and rods.^13^ The yellow complex is composed of different Streptococcus species.^13^ The green complex is composed of the Capnocytophaga species *Agregatibacter actinomycetemcomitans*, and Eikenella corrodens.^13^ The purple complex is composed by Actinomyces and Veillonella species.^13^ Although these three complexes are composed of microorganisms that colonize the resident microbiota in the gingival sulci, it is important to study the prevalence of such bacterial complexes in endodontic-periodontal lesions, since the imbalance in the number of these populations can lead to a pathogenic condition.^13^ In this environment, such microorganisms act ecologically, occupying their niches and forming a climax community.^9,13^ However, the transfer of elements of the resident microbiota from one anatomical site to another site where such organisms are not normally found can initiate a so-called as endogenous infection.^9,13^

The identification of the microbiological profile in endodontic-periodontal lesions is necessary to understand the pathogenesis and evolution of these lesions.^2^ Moreover, the targeting of new therapies for these lesions is also based on the microbial susceptibility of the involved species to the gold standard treatment (mechanical instrumentation) and to alternative/adjunctive treatments, such as the use of antimicrobials, host modulation therapy, photodynamic therapy, and probiotic therapy.^15-17^ A previous systematic review^9^ assessed the prevalence of species of red and orange microbial complexes in endodontic-periodontal lesions. However, the prevalence of yellow, purple, and green microbial complexes has not yet been evaluated. Thus, the purpose of this systematic review was to determine the prevalence of species of yellow, purple and green microbial complexes in endodontic-periodontal lesions.

## Methodology

### Registration and protocol

A systematic review protocol was previously registered in the Prospective International Registry of Systematic Reviews (PROSPERO)^18^ under the code CRD42020214414. The present review was reported according to the recommendations of the Preferred Reporting Items for Systematic Reviews and Meta-analysis (PRISMA 2020) statement.^19^

### Eligibility criteria

The PICOS strategy was used to define the eligibility criteria, as follows:

*Population*: permanent human teeth with endodontic-periodontal lesions;

*Intervention*: microbiological tests for bacterial profile identification;

*Comparison*: none, or permanent human teeth without endodontic-periodontal lesions;

*Outcome*: prevalence of bacterial species of the yellow, purple, and green complexes in periodontal pockets and root canals of teeth with endodontic-periodontal lesions;

*Study design*: clinical trials, cross-sectional studies and cohort studies.

The following exclusion criteria were applied: studies in immature teeth, reviews, case reports or case series, protocols, brief communications, personal opinions, letters, posters, conference abstracts, laboratory-based studies, and studies that did not investigate species of yellow, purple, or green microbial complexes.

### Information sources and search strategy

Electronic searches were conducted in the following electronic databases: Cochrane Library, Embase, Latin American and Caribbean Health Sciences (LILACS), MEDLINE PubMed, Scopus, and Web of Science. The grey literature was searched through Google Scholar, OpenGrey, and ProQuest dissertations & theses. To ensure literature saturation, the reference lists of the selected studies and reviews were manually checked to identify potentially relevant references that might have been missed during the initial search. All searches were conducted from the earliest date available until October 20^th^, 2020 and updated on January 3^rd^, 2022. No filters, limits, language, or publication date restrictions were applied. References were managed by a reference software (EndNote X7; Thomson Reuters, Philadelphia, PA).

### Selection process

The study selection process was performed in duplicate by two independent reviewers (D.J.G. and K.A.S.C.). First, potentially relevant studies were selected by reading the titles and abstracts. Then, the same reviewers separately applied the eligibility criteria during the full-text screening. Doubts or disagreements were resolved by an analysis of each study and discussion in the presence of the third reviewer (F.C.V.) to obtain consensus. At both times, a team of experts (J.P.C., R.R.M., B.P.F.A.G., and M.S.T.) cross-examined all the relevant information.

### Data collection process, data items and effect measures

A data collection form was previously developed and tested in a pilot study. After training, two reviewers (D.J.G. and K.A.S.C.) independently collected the critical data. Any disagreement was resolved between them and, if there was no consensus, a third reviewer (F.C.V.) was involved to steer the decision. Data extracted from each selected study included: study characteristics (authors, year, country, and design of study), sample characteristics (sample size, gender and mean age of participants), type of endodontic-periodontal lesion, detection method of the bacterial species, periodontal parameters (probing depth, mobility, bone loss, clinical attachment, and gingival bleeding), endodontic parameters (pulp status, symptomatology, and periapical lesion), and prevalence reports of bacterial species of the yellow, purple, and green bacterial complexes in root canals and periodontal pockets.

### Data analysis

The quantitative analysis (meta-analysis) of the pooled data was not possible due to the high methodological heterogeneity among the included studies regarding type of endodontic-periodontal lesion evaluated and microbiological tests applied to identify the species of microbial complexes. Therefore, data were analyzed qualitatively and presented in narrative form in order to address the scope and objectives of the present systematic review. All team members participated in this process for the best presentation of prevalence data.

### Risk of bias assessment

The risk of bias of the included studies was assessed using the NIH Quality Assessment Tool items for observational cohort and cross-sectional studies (https://www.nhlbi.nih.gov/health-topics/study-quality-assessment-tools). This tool covers 14 criteria. Response items are ‘yes’, ‘no’, ‘cannot determine’, ‘not applicable’, or ‘not reported’. All responses other than ‘yes’ indicate a possible risk of bias. Inherent to the design, cross-sectional studies automatically score ‘not applicable’ on criteria 6, 7, 10, and 13. Two reviewers (D.J.G. and K.A.S.C.) were previously trained and calibrated to use this tool by discussing each predetermined criterion. In case of disagreements, a third reviewer was involved to steer the decision. In the end, a calculation of these distributed scores was performed. Studies that scored ‘yes’ in up to 49% of the criteria were classified as ‘high risk of bias’; 50% to 69% as ‘moderate risk of bias’; and more than 70% as ‘low risk of bias’.

## Results

### Study selection and characteristics

The study selection process is summarized in Figure. The initial electronic search resulted in 1,611 references. After removing duplicates (n = 194), a total of 1,417 references were assessed in phase one (title and abstract reading). At the end of phase one, 12 studies were included for full-text evaluation. After full-text reading, eight studies were excluded (Table 1) and four^4,17,20,21^ studies were included in the systematic review.

**Figure:**
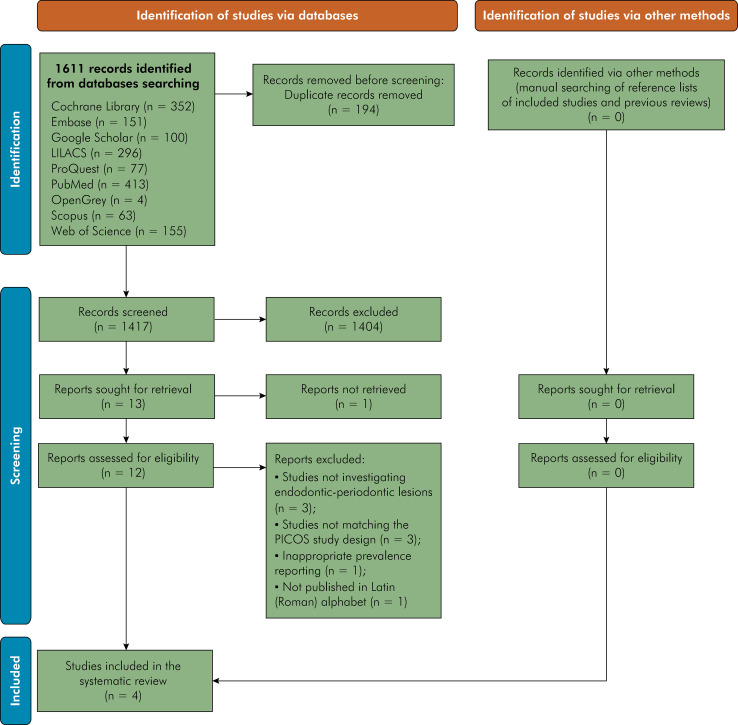
PRISMA flow chart: Overview of the study selection process.

**Table 1:** Studies excluded after the full-text reading and reasons for exclusion.

Reference	Reason for exclusion
Fujii R, Muramatsu T, Yamaguchi Y, Asai T, Aida N, Suehara M, Morinaga K, Furusawa M. An endodontic-periodontal lesion with primary periodontal disease: a case report on its bacterial profile. Bull Tokyo Dent Coll. 2014;55:33-7	Case report
Kurihara H, Kobayashi Y, Francisco IA, Isoshima O, Nagai A, Murayama Y. A microbiological and immunological study of endodontic-periodontic lesions. J Endod. 1995;21:617-21	Case series
Nielsen HK, Garcia J, Væth M, Schlafer S. Comparison of Riboflavin and Toluidine Blue O as photosensitizers for photoactivated disinfection on endodontic and periodontal pathogens in vitro. PLoS One. 2015;10:e0140720	Laboratory research
Reinhardt B, Klocke A, Neering SH, Selbach S, Peters U, Flemmig TF, Beikler T. Microbiological dynamics of red complex bacteria following full-mouth air polishing in periodontally healthy subjects-a randomized clinical pilot study. Clin Oral Investig. 2019;23:3905-3914.	Study not investigating endodontic-periodontic lesions
Rôças IN, Siqueira JF Jr. Culture-independent detection of Eikenella corrodens and Veillonella parvula in primary endodontic infections. J Endod. 2006;32:509-12	Study not investigating endodontic-periodontic lesions
Sassone LM, Fidel RA, Faveri M, Figueiredo L, Fidel SR, Feres M. A microbiological profile of unexposed and exposed pulp space of primary endodontic infections by checkerboard DNA-DNA hybridization. J Endod. 2012;38:889-93	Study not investigating endodontic-periodontic lesions
Xia M, Qi Q. Bacterial analysis of combined periodontal-endodontic lesions by polymerase chain reaction-denaturing gradient gel electrophoresis. J Oral Sci. 2013;55:287-91	Inappropriate prevalence reporting
Zhou K, Ji PH, Yu LY, Chen Q, Xu QL. [Detection of anaerobes and drug sensitivity from the periodontal pockets of patients with combined periodontal-endodontic lesions]. Shanghai Kou Qiang Yi Xue. 2013;22(1):72-6	Not published in the Latin (Roman) alphabet

Detailed information about the included studies is available in Table 2. The included studies had a cross-sectional design and were published between 2012 and 2020. The studies were conducted in Brazil,^21^ China,^17^ India,^4^ and Romania.^20^ The total sample consisted of 116 adult patients (age range 20 to 60 years) with teeth diagnosed with endodontic-periodontal lesions. All included studies evaluated one tooth from each patient, so the sample size of pooled teeth corresponded to 116. At least 46 molars, three premolars, and 57 single-rooted teeth were evaluated, given that one study^21^ did not report the type of evaluated teeth. Overall, the studies excluded patients with a history of antibiotic use between three and six months before the study; the presence of vertical root fracture, swelling, presence of sinus tracts, teeth with pulp chamber exposed to the oral cavity; active caries teeth; associated systemic disease; and patients undergoing periodontal treatment in the last 6 months of the study.

**Table 2:** Main characteristics of the included studies.

Authors, year	Country	Study design	Type of lesion	Patients (n)	Age range (mean)	Teeth	Detection method
Didilescu et al., 2012	Romania	Cross-sectional	N/I	46	N/I	17 single-rooted, three premolars and 26 molars	PCR and D-Dh
Li et al., 2014	China	Cross-sectional	I	20 (F: 6; M: 14)	29–60y (45.6y)	Only molars	PCR and DGGE
Rovai et al., 2019	Brazil	Cross-sectional	II	10	N/I	Not specified	D-Dh
Das *et* al., 2020	India	Cross-sectional	N/I	40 (F: 22; M: 18)	20–50y (F: 41.3y; M: 42.5y)	Only single-rooted teeth	PCR

(I) True combined injury lesion; (II) Primary periodontal lesions with secondary endodontic involvement; (D-Dh) DNA-DNA hybridization; (DGGE) Denaturing Gradient Gel Electrophoresis; (F) female; (M) male; (N/I) not informed; (PCR) polymerase chain reaction; (y) years.

Regarding the characteristics of the endodontic-periodontal lesions, ten teeth were diagnosed with primary periodontal lesions with secondary endodontic involvement, twenty teeth with true combined lesions, and 86 did not specify the classification of the lesions. All studies identified that teeth diagnosed with endodontic-periodontal lesions contained periodontal pockets ≥5 mm, evidence of periapical lesions on radiographic examination, and pulp necrosis. More detailed information about the periodontal and endodontic parameters is presented in Table 3. Qualitative and semiquantitative evaluation of bacteria profile was performed by DNA-DNA hybridization (D-Dh), polymerase chain reaction (PCR) and denaturing gradient gel electrophoresis (DGGE).

**Table 3:** Periodontal and endodontic parameters used in the included studies.

Study	Periodontal parameters						Endodontic parameters			

	Pocket depth	Mobility	Bone loss	Clinical	Gingival bleeding	Disease	Dental status	Pain	Pulpal status	Periapical lesion
Didilescu et al., 2012	> 5 mm	Yes	Yes	Yes	Yes	CLP	Healthy/ restored	Yes	Necrotic	Yes
Li et al. , 2014	≥ 7 mm	N/I	N/I	N/I	N/I	N/I	Healthy	N/I	Necrotic	Yes
Rovai et al., 2019	≥ 6 mm	N/I	N/I	N/I	Yes	N/I	Healthy	No	Necrotic	Yes
Das et al., 2020	≥ 6 mm	N/I	N/I	N/I	N/I	AP	Healthy	N/I	Necrotic	Yes

AP: Aggressive periodontitis; CLP: chronic and localized periodontitis; N/I: not informed.

### Risk of bias assessment

The interobserver agreement for the assessment of the risk of bias (*Kappa* = 0.872 [0.208]) was considered excellent.^22^ The risk of bias assessment is summarized in Table 4. None of the included studies met all of the NIH Quality Assessment Tool items. One study^20^ met eight items (57.14%) and three studies^4,17,21^ met seven items (50%). Thus, all included studies were classified as having ‘moderate risk of bias’.

**Table 4:** Risk of bias assessment by the NIH Quality Assessment Tool.

Criteria	Didilescu et al., 2012	Li et al., 2014	Rovai et al., 2019	Das et al., 2020
1. Research question	Yes	Yes	Yes	Yes
2. Study population	Yes	Yes	Yes	Yes
3. Participation fee of eligible people	Yes	Yes	Yes	Yes
4. Eligibility criteria	Yes	Yes	Yes	Yes
5. Sample size	Yes	Yes	Yes	Yes
6. Exposure assessment	N/A	N/A	N/A	N/A
7. Deadline	N/A	N/A	N/A	N/A
8. Exposure levels	C/D	C/D	C/D	C/D
9. Exposure measures	Yes	C/D	C/D	C/D
10. Repeat exposure assessment	N/A	N/A	N/A	N/A
11. Result measures	Yes	Yes	Yes	Yes
12. Blinding of evaluators	No	No	No	No
13. Follow-up fee	N/A	N/A	N/A	N/A
14. Statistical analysis	Yes	Yes	Yes	Yes
Total of ‘yes’ (%)	8 (57.14%)	7 (50%)		

C/D: cannot determine; N/A: not applicable; N/R: not reported.

### Prevalence of microbial complexes

Didilescu et al.^20^ investigated forty-six teeth presenting endodontic-periodontal lesions. Two species belonging to the green complex (*C. sputigena* and *E. corrodens*) were investigated. Only *C. sputigena* (4.3% of the root canals and 13% of the periodontal pockets) was identified. Li et al.^17^ investigated samples collected from twenty molars diagnosed with combined endodontic-periodontal lesions. Two bacteria belonging to the yellow complex were identified: *S. sanguinis* (15% of the root canals and 40% of the periodontal pockets) and *S. mitis* (30% of the root canals and 35% of the periodontal pockets). Two bacteria belonging to the green complex were identified: *C. granulosa* (10% of the root canals and 35% of the periodontal pockets) and *C. sputigena* (20% of the root canals and 30% of the periodontal pockets).

The study performed by Rovai et al.^21^ investigated the presence of bacteria belonging to the yellow (*S. gordonii, intermedius, mitis*, and *sanguinis*), purple (*A. odontolyticus* and *V. parvula*), and green (A. *actinomycetemcomitans, C. gingivalis, C. ochracea, C. sputigena*, and *E. corrodens*) complexes in ten teeth with endodontic-periodontal lesions. Only two bacteria species were identified: *V. parvula* (70% of the root canals and 50% of the periodontal pockets) and *C. sputigena* (70% of the root canals, not identified in periodontal pockets). Das et al.^4^ investigated samples from forty teeth with endodontic-periodontal pathology. Specimens of *A. actinomycetemcomitans* (green complex) were isolated from 12% of root canals and 58% of periodontal pockets.

Considering the results of the four included studies, the profile and prevalence rates of bacterial species in root canals and periodontal pockets were heterogeneous. Levels of seven species were identified: *A. actinomycetemcomitans* (12% in root canals, 58% in periodontal pockets), *C. granulosa* (10% in root canals, 35% in periodontal pockets), *C. sputigena* (15 to 70% in root canals, 0 to 30% in periodontal pockets), *S. mitis* (30% in root canals, 35% periodontal pockets), *S. sanguinis* (30% in root canals, 35% in periodontal pockets), and *V. parvula* (70% in root canals and 50% in periodontal pockets).

## Discussion

Endodontic-periodontal lesions pose a challenge to the clinician in terms of diagnosis and therapeutic approach, which may reflect an unfavorable prognosis for the affected teeth if not properly treated.^2^ In these lesions, bacterial infection influences the clinical course of the disease and the treatment plan.^20^ The main purpose of this review was to determine the prevalence of species of the yellow, purple, and green microbial complexes in periodontal pockets and root canals of teeth with endodontic-periodontal lesions.

Studies on endodontic-periodontic lesions usually mention five bacterial complexes, first described by Socranski et al.^13^ including the yellow, purple, green, red, and orange. The red complex is the most commonly investigated due to its high pathogenicity; the orange complex can also have a high prevalence and may prevent the colonization of species from the red complex; the yellow complex has primary microorganism that allow the transition to the pathogenic complexes; the purple complex has only two species that are closely related to each other; and the green complex has bacteria that are able to adhere to the pellicle by means of fimbriae and thereby prevent them being flushed out with the gingival crevicular fluid.^1,2,14^

Bacteria from the green, purple, and yellow complexes are named “early colonizers” because they can adhere to the pellicle^1^ and have been associated with periodontal health.^14^ However, studies have shown that a large number of putative virulence factors were upregulated in a set of organisms not typically associated with periodontitis, such as S. oralis, S. mutans, S. intermedius, *S. mitis, V. parvula*, and P. fluorenscens, some of which are often associated with periodontal health.^14,23^ Therefore, the inflammation and tissue destruction in periodontitis is not only due to the presence of some periodontopathogens such as bacteria from the red complex, but to the dysbiosis of the oral microbiota that they induce.^24^ While the microbial composition of subgingival biofilm differs in healthy individuals, the development of periodontitis is directly related to dysbiosis, which is a shift from beneficial symbiotic bacteria to pathogenic bacteria.^24^ This shift triggers the potent host inflammatory response that contributes to tissue destruction and alveolar bone loss that are characteristic of periodontitis.^24^ The host’s immune response is dysregulated either because it is disrupted by the microbial community or due to host immunoregulatory defect, resulting in bacterial outgrowth and overt pathogenicity.^25^ Moreover, microbial dysbiosis has effects on human physiology that go beyond periodontitis and involve a number of clinically important processes, such as obesity, colitis, inflammatory bowel disease, and colorectal cancer.^24^

Overall, the most prevalent bacterial species of the yellow, green, and purple microbial complexes in periodontal samples were A. *actinomycetemcomitans, V. parvula*, and *S. sanguinis* and in root canals samples were *V. parvula, C. sputigena*, and *S. mitis. V. parvula* - belonging to the purple complex – is a gram-negative anaerobic coccus.^16^ In general, Veillonella species are characterized as incapable of fermenting sucrose and carbohydrates, depending on the excretion of organic acids by other species to obtain their energy.^16^ Streptococcus species release acids from their fermentation capacity, such as lactic acid, which is metabolized and used as an energy source by Veillonella species.^16,26^ In isolation, *V. parvula* is considered a beneficial bacterium of the oral biofilm - its presence in the subgingival plaque can contribute to the improvement of periodontal health.^27^ Rovai et al^21^ reported a high prevalence of *V. parvula* in root canals and periodontal pockets of teeth with endodontic-periodontal involvement. More clinical studies should be performed to identify the presence of this species and elucidate whether it plays a role in the occurrence and development of these lesions.

*S. mitis* is a facultative anaerobic gram-positive coccus that is considered abundant in the healthy human oral microbiota, and its decrease or elimination influences periodontal health status.^15^ It is considered a commensal species responsible for the initial colonization of dental biofilm.^28^ Despite not having a direct harmful effect, this bacterium can co-aggregate with other pathogenic bacteria - such as F. nucleatum and A. naeslundii - and therefore play a relevant role in the periodontal biofilm formation.^29^ Previous studies also demonstrated that the Streptococcus species was one of the most abundant and prevalent species in root canals.^30,31^

*C. sputigena* was a bacterium with high prevalence both in root canals and in periodontal pockets. It is a gram-negative, spindle-shaped, capnophilic bacillus found in the normal flora of the throat.^32^ In dental biofilm, *C. sputigena* is considered a beneficial microorganism; it is uncommon in endodontic infections but can be identified in cases of unexposed pulp tissue.^33^ Although it is not considered aggressive, the increased growth of this bacterium is thought to be involved in forms of destructive periodontal disease.^33^ Therefore, the presence of this bacterium in the pathogenesis of endodontic-periodontal lesions should be further investigated.

Endodontic-periodontal infections are typically polymicrobial, therefore synergistic and antagonistic interactions between different species are expected.^20^ This seems to be a factor that explains the low prevalence of *A. actinomycetemcomitans* found in the present review. A previous study^34^ demonstrated that *A. actinomycetemcomitans* suffers from growth limitations when in the presence of lactic acid bacteria, such as Veillonella species. Thus, *A. actinomycetemcomitans* and Veillonella seem to compete for the same nutrient, and the nutritional preference of one species can influence the existence of the other. Therefore, it seems to be expected that the higher prevalence of *V. parvula* results in a lower prevalence of *A. actinomycetemcomitans* in periodontal pockets and root canals.

The test used to identify bacterial species varied between included studies: D-Dh,^20,21^ DGGE^17^ and PCR.^4,17,20^ Despite these tests being originally designed to detect periodontal pathogens, they can be used in endodontic samples, assuming that the number of bacterial cells in an endodontic infection usually ranges from 10^3^ to 10^8^.^35^ Although they are different tests, they all use molecular biology techniques for bacterial identification.^36^ Molecular biology techniques have been widely used for the identification of oral microorganisms since microbiological culture methods have limitations such as the difficulty or unfeasibility of cultivating some species, greater demand for culture media and laboratory conditions, need to control microbial reproduction, and proper transport of samples.^36^ Therefore, microbiological culture may fail to detect pathogenic species involved in the evaluated disease.^37^

D-Dh molecular method is a quick and sensitive technique, which allows the identification of cultivable and non-cultivable bacterial species through the use of probes containing genomic DNA of the species to be investigated.^33^ This technique has been increasingly used to identify the endodontic and periodontal microbiota, combined with the PCR technique to confirm the DNA of the detected species.^33^ However, the impossibility of detecting species in the absence of prepared probes and the presence of microorganisms below the detection limits of the method (10^3^ to 10^4^) are some of its main limitations.^33^ In the present systematic review, the included study that addressed PCR/D-Dh was responsible for a greater detection of species of the yellow and green bacterial complexes.^20^

The conventional PCR technique using universal primers allows the amplification of an informative portion of the coding gene for 16S ribosomal RNA. The resulting amplicons can be sequenced and the exact composition of the microbial community can be accessed in qualitative terms by comparison of sequence data against a library of genome data.^31^ However, unless quantitative methods such as real-time PCR are applied, the exact amount of each of the community members cannot be determined.^20,31^ Checkboard DNA-DNA hybridization is a semi-quantitative technique that can be used to determine the relative proportion between members by the intensity of the signal emitted by the hybridization of total DNA from the microbial community sample against the probes immobilized on a matrix (e.g. a DNA microarray slide).^31^

Endodontic-periodontal lesions have the dental biofilm as an etiological factor, which is modulated by microorganisms, including bacteria.^4^ Streptococcus, Campylobacter, Eubacterium, Prevotella, and Fusobacterium species usually dominate endodontic infections.^20^ However, in teeth that also have periodontal involvement, the root canal microbiota changes - previous studies that investigated the profiles of pathogens in endodontic-periodontic lesions noticed a regular occurrence of periodontal pathogens in root canal infections.^11,12,17,20^ Microorganisms from the red and orange microbial complexes - known as “periodontal pathogens” - have been commonly isolated from root canal infections in teeth with endodontic-periodontal involvement, suggesting that periodontal pockets are a source of infection in the root canals.^11,12^ However, few studies investigate the presence of yellow, green, and purple microbial complexes in periodontal pockets and root canals of teeth diagnosed with endodontic-periodontal lesions. Qualitative and quantitative studies of these pathogens should be encouraged in the literature to increase this field of knowledge.

Endodontic-periodontal lesions tend to be classified according to the order in which the pathological processes originate and evolve in the pulp and periodontal tissues.^7^ In this systematic review, the classical classification proposed by Simon and colleagues^5^ - based on the origin of the lesions – was used. Of the four included studies, one study^21^ classified the lesions as primary periodontal lesions with secondary endodontic involvement, one study^17^ classified as a true combined lesion, and two studies^4,20^ classified as endodontic-periodontal lesions in general, without specifying the classification. This factor highlights a limitation of the included studies and affects the indirect evidence of the present review; therefore, the extrapolation of the results should be considered with caution by clinicians.

The four studies included in this systematic review were assessed as having ‘moderate’ risk of bias. Considering that four items cannot be evaluated in NIH Quality Assessment Tool items due to the nature of the study - cross-sectional - the general performance of the studies was positive. The main limitation concerns the absence of evaluator blinding, where all studies were evaluated as ‘high risk of bias’. Certainly, the fact that the included studies collected exposure and outcome data simultaneously limits the strength of the evidence of the present review. However, due to the objective of these studies - to assess the prevalence of microbial complexes – and their cross-sectional characteristic, the results produced can serve as a basis for follow-up studies that will certainly benefit from these data.

Regarding the methodological limitations of this systematic review, it can mention the low number of articles that investigated the outcome of interest and were included, as well as their cross-sectional design. In addition, the methodological heterogeneity used to detect microorganisms resulted in unequal quantification of bacterial species. To improve the quality of the evidence in this field of study, it is important that future clinical studies be conducted with greater methodological rigor, using larger and representative samples, subgrouping patients according to endodontic-periodontal lesion type, and standardizing the methodological steps and the microbiological methods used for bacterial identification and quantification.

## Conclusion

The results of the studies included in this systematic review suggest that the profile and prevalence of species belonging to the yellow, purple, and green complexes in periodontal pockets and root canals were not similar. Species of A. actinomycetemcomitans, *C. granulosa, C. sputigena, S. mitis, S. sanguinis*, and *V. parvula* were identified in both sites, but with different prevalence rates. Future clinical studies are encouraged to investigate the presence and role of these species in the occurrence and development of endodontic-periodontal lesions.
